# Generation and analysis of expressed sequence tags from the ciliate protozoan parasite *Ichthyophthirius multifiliis*

**DOI:** 10.1186/1471-2164-8-176

**Published:** 2007-06-18

**Authors:** Jason W Abernathy, Peng Xu, Ping Li, De-Hai Xu, Huseyin Kucuktas, Phillip Klesius, Covadonga Arias, Zhanjiang Liu

**Affiliations:** 1 The Fish Molecular Genetic and Biotechnology Laboratory, Department of Fisheries and Allied Aquacultures and Program of Cell and Molecular Biosciences, Aquatic Genomics Unit, Auburn University, AL 36849 USA; 2 Aquatic Animal Health Research Laboratory, Agricultural Research Service, United States Department of Agriculture, Post Office Box 952, Auburn, AL 36831 USA

## Abstract

**Background:**

The ciliate protozoan *Ichthyophthirius multifiliis *(Ich) is an important parasite of freshwater fish that causes 'white spot disease' leading to significant losses. A genomic resource for large-scale studies of this parasite has been lacking. To study gene expression involved in Ich pathogenesis and virulence, our goal was to generate expressed sequence tags (ESTs) for the development of a powerful microarray platform for the analysis of global gene expression in this species. Here, we initiated a project to sequence and analyze over 10,000 ESTs.

**Results:**

We sequenced 10,368 EST clones using a normalized cDNA library made from pooled samples of the trophont, tomont, and theront life-cycle stages, and generated 9,769 sequences (94.2% success rate). Post-sequencing processing led to 8,432 high quality sequences. Clustering analysis of these ESTs allowed identification of 4,706 unique sequences containing 976 contigs and 3,730 singletons. These unique sequences represent over two million base pairs (~10% of *Plasmodium falciparum *genome, a phylogenetically related protozoan). BLASTX searches produced 2,518 significant (E-value < 10^-5^) hits and further Gene Ontology (GO) analysis annotated 1,008 of these genes. The ESTs were analyzed comparatively against the genomes of the related protozoa *Tetrahymena thermophila *and *P. falciparum*, allowing putative identification of additional genes. All the EST sequences were deposited by dbEST in GenBank (GenBank: EG957858–EG966289). Gene discovery and annotations are presented and discussed.

**Conclusion:**

This set of ESTs represents a significant proportion of the Ich transcriptome, and provides a material basis for the development of microarrays useful for gene expression studies concerning Ich development, pathogenesis, and virulence.

## Background

The ciliate protozoan *Ichthyophthirius multifiliis *(Ich)is one of the most devastating pathogens. It infects fish skin and gills, and causes white spot diseases in many species of freshwater fish worldwide, which leads to significant losses in the aquaculture industry. The ciliate parasite has three main life-cycle stages: the reproductive tomont, the infective theront, and a parasitic trophont [1-3]. The mature trophont drops off the host to become the tomont where it attaches to a substrate, and undergoes multiple divisions to produce hundreds to thousands of tomites within a cyst. Tomites bore their way through the cyst into water, and differentiate into theronts that infect fish. Once they burrow into the fish epithelium, theronts become trophonts that feed and mature in the host.

In spite of great losses caused by Ich to the aquaculture industry, molecular studies of the parasite have been scarce [see a recent review [4]]. Limited studies have concentrated on immune responses of the host and factors affecting them [5-11]. One of the difficulties for the studies of Ich is the problem involved in long-term maintenance of Ich isolates. Ich isolates appear to lose infectivity or become senescent after a certain number of passages [12-15]. Most often a significant decrease in infectivity is observed after about 50 passages [15]. Not only the infectivity decreases with higher numbers of passages, but also the development of the parasite as measured by the period required for trophonts to emerge from fish [15].

The Ich senescence phenomenon is interesting not only as a developmental biology issue, but also as a potential research system to study the virulence factors involved in the parasite pathogenesis. Assuming the life cycles of Ich and its infectivity are controlled by gene products, then it would be of great interest to learn what genes are involved in the loss of infectivity, and in the slowing down of its development. However, as very limited molecular information is available from Ich, in-depth research is limited by the lack of information and the lack of genomic resources.

EST analysis is one of the most effective means for gene discoveries, gene expression profiling, and functional genome studies [16-23]. It is also one of the most efficient ways for the identification of differentially expressed genes [24-28]. In order to provide genomic resources for the analysis of differentially expressed genes at different developmental stages of the Ich parasite, and for the analysis of genes differentially expressed when infectivity is being lost, the objectives of this study were to create cDNA libraries suitable for the analysis of expressed sequence tags (ESTs) and to generate an EST resource for Ich to allow cDNA-based design of microarrays for the study of gene expression in relation to the passages and development of the parasite. Before this work, there were only 511 Ich sequences in the GenBank dbEST (release 100606) [29]. A brief examination of these existing EST sequences indicated that a large proportion of them were trophont only reads, histones, ribosomal proteins, and immobilization antigen-related sequences. Here we report sequencing of 10,368 Ich EST clones, and generation of 8,432 high quality EST sequences. This EST resource should provide the material basis for the development of microarrays for Ich, and serve as a platform for its functional genomic studies including the development and pathogenesis of Ich, and the host-parasite interactions.

## Results and Discussion

### Generation of the Ich ESTs

As summarized in Table 1, a total of 10,368 clones were sequenced from a normalized Ich library made from pooled cells from all three life cycle stages: theront, tomont, and trophont. Readable sequences were generated with 9,769 clones (94.2% sequencing success rate). After base calling, sequences were processed by using Phred [30,31] to eliminate low quality sequences below Q20. Sequences passing Q20 were uploaded into Vector NTI Advance 10 (Invitrogen, Carlsbad, CA) for vector trimming and removal of sequences with very short inserts (<100 bp). The post-sequencing processing resulted in 8,432 high quality sequences.

**Table 1:** A summary of the EST analysis.

Description	Number	Percentage
Total number of clones sequenced	10,368	
Total number of successful sequences	9,769	94.2%
Number of high quality sequences	8,432	86.3%^1^
Unique sequences	4,706	55.8%^2^
Number of contigs	976	
Number of clones included in the contigs	4,702	
Average clones per contig	4.82	
Number of singletons	3,730	
Number of known genes	2,518	53.5%^3^
Unique unknown genes	2,188	46.5%^3^

^1^Percentage of high quality sequences from successful sequences; ^2^percentage of unique sequences of the high quality sequences; ^3^percentage of unique sequences

The processed sequences were subjected to cluster analysis using Vector NTI to evaluate sequence redundancies. Of the 8,432 sequences, 4,702 sequences fell within 976 contigs while 3,730 sequences were singletons. On average, each contig contained 4.8 sequences. Taken together, the 976 contigs and the 3,730 singletons made up 4,706 unique sequences (Table 1).

The Ich genome expression appeared to be extremely polarized with a few genes expressed at very high levels. In spite of normalization, transcripts from a few genes were sequenced at very high frequencies. The top 20 contigs with the largest number of ESTs are summarized in Table 2. Of the top 20 most abundantly sequenced transcripts, four of them were detected over 0.5% of total sequences. Of these, the most abundantly sequenced EST cluster, cluster 276 with 764 ESTs, accounted for 7.36% of all sequenced clones. BLASTX searches indicated that this transcript was most similar to a hypothetical protein, TTHERM_02141640, from *Tetrahymena thermophila*. The second most abundantly sequenced transcript was cluster 60 with 119 ESTs. It was identified as a transcript most similar to a hypothetical protein, TTHERM_02641280, from *T. thermophila*. The functions of these hypothetical proteins are unknown at present. These are two transcripts sequenced at exceptionally high frequencies. Obviously, the presence of such abundant transcripts suggested a failure in the normalization processes. However, it is puzzling to us because we believe the overall normalization processes may have worked based on several other observations: 1) the overall gene discovery rate (unique sequences over all sequences analyzed) was 55.8%, a reasonable rate for the sequencing depth of approximately 10,000 clones; 2) most other anticipated highly expressed genes such as ribosomal protein genes, actin genes, tubulin genes, and dynein genes were not detected at high levels. Nonetheless, we believe that this information is relevant and important as these genes should be the subject for additional subtraction for further EST sequencing in this species. In addition, such information should provide some basic picture about the most abundantly expressed genes in the parasite. As these hypothetical protein genes are transcribed at such high levels, they may be crucially important for the growth and development, or other life-cycle processes of the parasite.

**Table 2:** The most abundant ESTs detected from the EST sequencing

Cluster	# of Sequences	Putative identities	% of Total
276	764	Hypothetical protein TTHERM_02141640 from *Tetrahymena thermophila*	7.36%
60	119	Hypothetical protein TTHERM_02641280 from *Tetrahymena thermophila*	1.15
636	86	Unknown	0.82
602	78	Unknown	0.75
171	48	Heat shock protein 90	0.46
83	39	Zinc finger ZZ type family protein	0.38
105	38	Unknown	0.37
279	35	Heat shock protein 90	0.34
392	34	Unknown	0.33
354	31	Heat shock protein 70 (dnaK)	0.30
203	31	Conserved hypothetical protein from *Paracoccus denitrificans*	0.30
219	29	Hypothetical protein PY05925 from *Plasmodium yoelii*	0.28
932	28	Unknown	0.27
75	27	Unknown	0.26
472	24	ER type HSP70	0.23
351	23	Unknown	0.22
833	23	Unknown protein from *Oryza sativa*	0.22
131	22	Unknown	0.21
6	21	Dynein heavy chain protein	0.20
45	21	Outer surface protein from *Rickettsia typhi*	0.20

This work demonstrated that pooling of samples from all three stages of Ich life cycle followed by normalization was an effective way to reduce common messages across all three life stages. As one would expect, many structural genes would be expressed highly abundantly in all stages of the life cycle. In addition to making savings economically, pooling of samples allowed very effective normalization of these common transcripts without going through three rounds of normalization. This is consistent with our previous experience for the generation of a large number of catfish and oyster ESTs [32-36]. It is obvious that the pooling of samples from three developmental stages made it impossible to provide information concerning expression profiling in relation to developmental stages. However, such information would not be highly meaningful in normalized cDNA libraries where the major focus was to develop EST resources, rather than expression profiling. The other limitation caused by construction of a pooled cDNA library is the loss of sequencing flexibility as to the number of clones to be sequenced from each developmental stage library if they had been separately constructed.

The Ich transcribed sequences are highly A/T-rich, similar to the situation in *T. thermophila*. Our unique sequences combined contain 2.18 megabases, approximately 10% of the genomic sequence size of the related protozoan *Plasmodium falciparum*, and 2.1% of the *T. thermophila *genomic sequence. As Ich is a ciliate and most closely related to *Tetrahymena*, this EST resource should represent a good sample of the transcribed fraction of the Ich genome for the estimation of its genome contents as compared with *Tetrahymena*. Based on the EST sequences, the average G+C content of Ich transcribed sequences was found to be 33.4%, even more A/T-rich than those of the closely related hymenostome *T. thermophila*, which has an average G+C content of 38% at protein coding regions [37]. The entire genome of *T. thermophila *was much more A/T-rich than the transcribed fraction, with a G+C content of only 22% [38]. It is highly probable that the Ich genome is also highly A/T-rich. To further the analysis, we found approximately 1% of the unique ESTs sequenced contained simple sequence repeats. The majority of the simple sequence repeats were of di-nucleotide repeats (68.8%) with AC and AG repeats being the majority. Tri-nucleotide and tetra-nucleotide repeats accounted for 23.7% and 7.5% of the identified microsatellites, respectively (Table 3).

**Table 3:** A summary of simple sequence repeats identified from the Ich ESTs. Percentages indicated in the parentheses are percentage of each type of repeat among all repeats

Total number of sequences analyzed	8,432
Number of dinucleotide repeats	422 (68.8%)
Number of AC repeats	121
Number of AG repeats	108
Number of AT repeats	56
Number of CT repeats	49
Number of GT repeats	88
Number of GC repeats	0
Number of trinucleotide repeats	145 (23.7%)
Number of tetranucleotide repeats	46 (7.5%)
Total number simple sequence repeats	613

The putative identities of the sequenced ESTs were assessed using BLASTX searches against the non-redundant (NR) database in GenBank [39]. All the search results are summarized in supplemental Table 1. Of the 4,706 unique ESTs, 2,518 (53.5%) had significant (E-value < 10^-5^) hits. The remaining 2,188 (46.5%) EST sequences were not similar to any known sequences. Additional searches using the Swiss-Prot database resulted in putative identities for six additional unknown ESTs (Supplemental Table 1).

### Identification of putative secretory proteins

Secretory proteins have been shown to be an important component in many biological processes, including pathogenesis of parasites [40-42]. We therefore searched for transcripts with putative signal peptides (suggestive of peptides of secretory proteins) within the EST set using the program SignalP 3.0 [43]. We found 314 ESTs with signal peptides, representing 6.7% of the unique sequences. Of these, 180 (3.8%) were from ESTs with no significant (E-value < 10^-5^) BLASTX hit to the NR database in GenBank (Supplemental Table 1).

### Comparative analysis to related taxa

The parasite Ich is phylogenetically placed between the protozoan's *Plasmodium falciparum *and *Tetrahymena thermophila*. Previous studies using 18S rDNA, histone genes, and I-antigens [44-46] suggested that Ich was more related to *T. thermophila *than to *P. falciparum*. Furthermore, *T. thermophila *and Ich share the ciliate nuclear genetic code, while *P. falciparum *uses the standard genetic code for translation. As the entire genome sequence of *P. falciparum *is available and the macronuclear sequencing project of *T. thermophila *was just recently completed, we made comparative BLAST analyses against both genome sequences.

**Figure 1:**
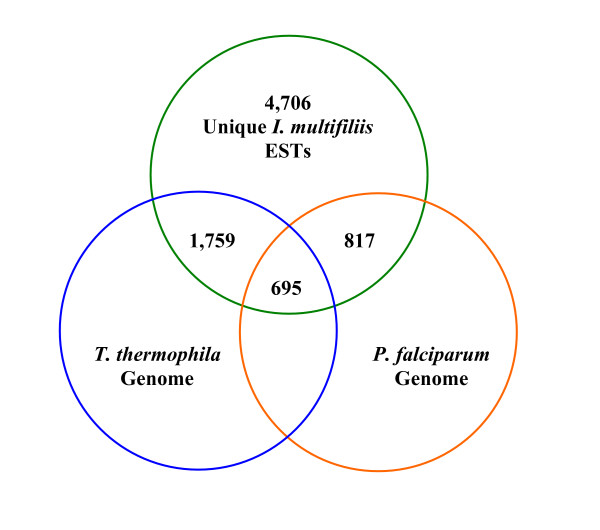
Venn diagram summary of sequence comparisons of the Ich ESTs with *Tetrahymena thermophila *and *Plasmodium falciparum *genomes. A total of 4,706 unique Ich ESTs were used as queries yielding 1,759 significant (E-value < 10^-5^) hits to the *T. thermophila *genome, and 817 to the *P. falciparum *genome. A total of 695 sequences were ESTs with common hits to both genomes.

The tBLASTx or BLASTX searches of Ich ESTs against the *T. thermophila *and *P. falciparum *genomes are summarized in Supplemental Table 2, and are presented in Figure 1. As expected based on the phylogenetic relationships, more Ich ESTs were similar to the genome sequences of *T. Thermophila *than to that of *P. falciparum*. Of the 4,706 Ich ESTs, 1,759 sequences were similar (E-value < 10^-5^) to the *T. thermophila *genome sequences; whereas 817 were similar to the *P. falciparum *genome sequences. In total, 695 ESTs were similar to both *T. thermophila *and *P. falciparum *genomes, and thus are common to all three protists.

Of the 1,759 significant hits against the *T. thermophila *genome, 1,673 had been identified with a putative identity using BLASTX searches against the NR database, while the tBLASTx searches against the *T. thermophila *genome allowed identification of putative identities for additional 86 unique ESTs. Similarly, BLASTX searches against the *P. falciparum *genome allowed identification of 9 additional ESTs. Taken together, the BLAST searches against these two genomes allowed putative identities of 95 additional unique ESTs, bringing the total number of ESTs with significant similarities to known genes to 2,613.

Such genome searches also revealed that of the 2,518 ESTs that had significant hits in BLASTX searches against the NR database, 845 had no significant hits to the *Tetrahymena *genome. Clearly, these ESTs were similar to sequences of organisms other than the ciliate *Tetrahymena*.

These results clearly suggest conservation of a large fraction of gene sequences among the three protozoa parasites, with a higher level of conservation between Ich and the *T. thermophila *genome than between the Ich genome and the *P. falciparum *genome; although a significant fraction of gene sequences are also shared between the genomes of *T. thermophila *and *P. falciparum*. The results of this comparative analysis are compatible with existing phylogenetic analyses using several molecular markers such as the 18S rDNA, histone genes, and the I-antigens. Obviously, use of a large set of sequences should provide a greater confidence concerning genome evolution. The comparative analysis suggested that the EST resource generated from this study should be useful for phylogenetic analysis and studies concerning genome evolution.

### Gene ontology

The unique Ich sequences were compared to annotations through the Gene Ontology Consortium [47] using the automated software Blast2GO [48]. We were able to obtain GO terms for 1,008 unique sequences using this method. Of these, 304 were contigs and 704 were singletons. Sequence descriptions, gene ontology (GO) and enzyme commission (EC) numbers are summarized in Supplemental Table 3. There were 258 sequences with both GO terms and EC numbers.

**Figure 2:**
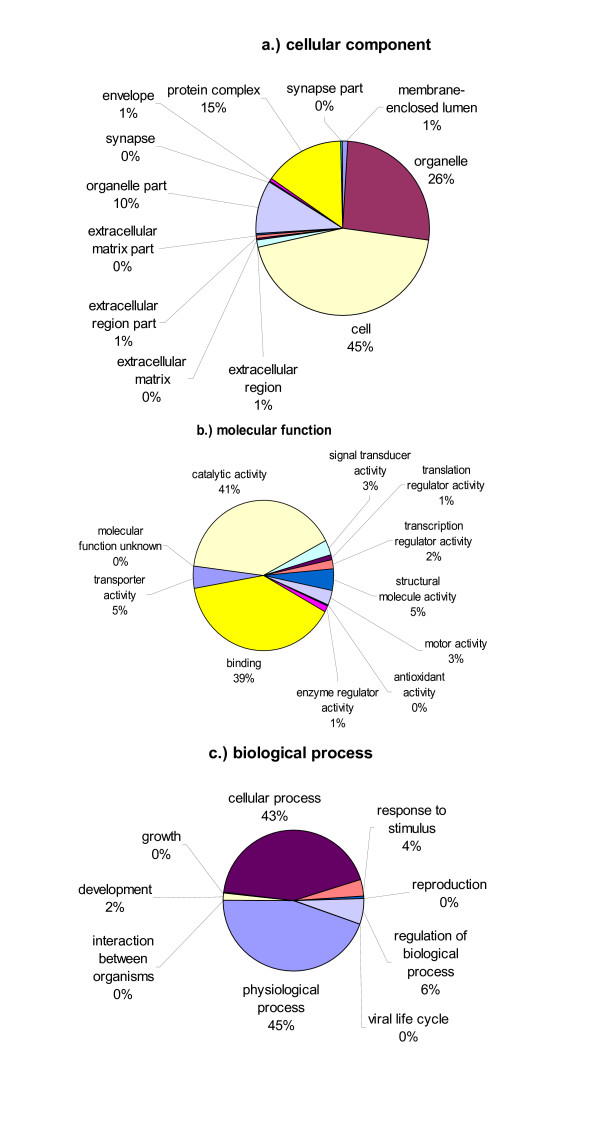
Pie charts of 2^nd ^level gene ontology (GO) terms. Overall, 1,008 unique sequences were annotated using the Blast2GO software and included in the graphs. Each of the three GO categories is presented including cellular component (a), molecular function (b), and biological process (c).

Gene ontology graphs using percentages of 2^nd ^level GO terms are presented in Figure 2 under the categories of cellular components (Fig. 2A), molecular functions (Fig. 2B), and biological processes (Fig. 2C). Of the cellular component GO terms, 45% and 26% were related directly with cellular and organelle components, respectively. In the category of molecular functions, the vast majority were involved in catalytic activity (41%) and binding activities (39%). Under the category of biological processes, 45% were involved in physiological processes; 43% were involved in cellular processes, 6% in regulation of biological processes, 4% in response to stimuli, and 2% in development (Figure 2).

## Conclusion

We have produced 8,432 high quality *I. multifiliis *EST sequences. Sequence analysis indicated the presence of 4,706 unique sequences in the EST set. This should represent a significant fraction of the Ich genes, although the exact gene number of the parasite is unknown at present. The majority of the unique EST sequences had similarities to known genes, making them more amenable to functional analysis. The EST sequences should enhance the effectiveness of molecular studies, especially for gene expression profiling and the analysis of genes involved in virulence and infectivity. Microarrays can now be designed using either cDNA microarray or oligo-based platforms using the EST information. Additionally, the cluster and redundancy information should be useful for further subtraction of the most abundant transcripts included in the cDNA library, making further EST analysis in the parasite more effective.

## Methods

### Samples

The source of mRNA for this analysis was derived and expanded from a single parasite cloned from the infected fish. The source of the original *Ichthyophthirius multifiliis *was isolated from an infected fish obtained from a local pet shop and the parasite was transmitted to channel catfish held in a 50-l glass aquarium at the USDA-ARS Aquatic Animal Health Research Laboratory, Auburn, AL. The transmission of *I. multifiliis *was achieved through co-habitation of the infected fish with two fingerlings of channel catfish (3 inches in size). When the two catfish were infected, the symptoms of Ich, white spots, started to emerge when trophonts were collected by scraping with a glass slide. Channel catfish infected with maturing trophonts were rinsed in dechlorinated water and the skin was gently scraped to dislodge the parasites. Trophonts were harvested by filtering through a 0.22 *μ*m filter to remove fish skin. The trophonts were placed into a Petri dish to allow them to develop into theronts that were used to infect 8 fish each for the collection of trophonts, toments, and theronts, respectively. Trophonts were directly collected from the skin surface of the 8 infected fish. To collect tomonts and theronts, trophonts isolated from fish were placed in Petri dishes and allowed to attach. After replacing the water in the Petri dishes with fresh dechlorinated water to remove contaminating mucus, the trophonts were incubated at 24°C for 8 h to harvest tomonts (32–128 cells/cyst) or 24 h to harvest theronts. Trophonts, tomonts and theronts were washed with PBS (pH 7.4), concentrated with a centrifuge (Beckman Coulter, Inc., Miami, FL) at 228 × g for 5 min and discarded supernatant. After washing 3 times with PBS, parasite samples from the three life stages were stored in liquid nitrogen and used for the isolation of RNA for the construction of normalized cDNA library.

### RNA isolation

Total RNA was isolated from the samples using the TRIzol reagent method from Invitrogen (Carlsbad, CA, USA) according to manufacturer's instructions. Briefly, samples of tomont, theront, and trophont were resuspended after thawing on ice, and 100 μl each were combined in a sterile tube to provide a total of 300 μl of Ich samples with equal fractions from each of its three life stages. As the major objective of this study was to generate EST resources with maximal efficiency of gene discovery, a pooled sample followed with normalization would allow inclusion of all transcripts in the library while reducing cost for library construction and increasing gene discovery rate. Three milliliters of TRIzol reagent was added to the sample tube. Cells were lysed by repetitively pipetting up and down. RNA was isolated following the manufacturer's protocol. The RNA pellet was resuspended in 100 μl of RNase-free double distilled water and divided into 25 μl aliquots. RNA aliquots were checked for quality using agarose gel electrophoreses containing formaldehyde.

### Normalized library construction

The Creator Smart cDNA Library Construction Kit from Clontech (Mountain View, CA) and components from the TRIMMER-DIRECT Kit from Evrogen (Moscow, Russia) were used for the construction of the normalized cDNA library. Total RNA concentration was checked on a spectrophotometer and 1 μg RNA was combined with 1 μl of SMART IV oligonucleotide (Clontech) and 1 μl CDS-3M adapter (Evrogen) for first-strand cDNA synthesis. The reaction was incubated at 72°C for 2 min followed by immediate cooling on ice for 2 min. Next, 2 μl of 5× first strand buffer, 1 μl of DTT (20 mM), 1 μl of dNTP mix (10 mM), and 1 μl of PowerScript reverse transcriptase were added to the tube and incubated at 42°C for 1 h in a thermal cycler (PTC-100, Bio-Rad, Hercules, CA) then placed on ice. The SMART cDNA cloning system allows the enrichment of full-length cDNA through the use of a 5'-linker with 3'-GGG tails. Reverse transcriptase has terminal transferase activity that preferentially adds three additional Cs at the end of first strand cDNA. As a result, the first strand cDNA is able to base pair with the 5'-linker with 3'-GGG tails. Once base paired, the reverse transcriptase would switch the template and extend into the linker sequences allowing PCR amplification of full-length cDNA using a single primer (the 5'-linker has the same sequences as the linker containing poly T used for the synthesis of the first strand cDNA). Truncated cDNAs are not able to base pair with the 5'-linker and, therefore, get lost in the PCR amplification of the full-length cDNA.

The first strand cDNA was initially amplified by long-distance PCR (LD-PCR) using hot-start amplification. For the reaction, the following were combined in a reaction tube: 1.5 μl of the first strand cDNA, 60 μl of sterile deionized water, 7.5 μl of 10× Advantage 2 PCR buffer, 1.5 μl of 50× dNTP mix, 3 μl of 5' PCR primer and 1.5 μl of 50× Advantage 2 polymerase mix. The tube was mixed and briefly centrifuged and added to a pre-heated (95°C) thermal cycler. Cycle settings were 95°C for 1 min followed by 19 cycles of 95°C for 7 s, 66°C for 20 s, and 72°C for 5.5 min. The product was analyzed on a 1.1% agarose gel to determine the sizes and amount of the cDNA products before proceeding to the next step. The LD-PCR reaction was purified and eluted in 30 μl of sterile Nanopure water using the QIAquick PCR Purification Kit (Qiagen, Valencia, CA).

For the normalization procedure, the TRIMMER-DIRECT Kit from Evrogen (Moscow, Russia) was used. This system is specially developed to normalize cDNA enriched with full length sequences [49,50]. The cDNA from the LD-PCR was quantified [~100 ng/μl] and 1 μl was mixed with 1 μl of the 4× hybridization buffer and 2 μl of sterile water. The mix was overlaid with mineral oil and incubated for 3 min at 98°C followed by 4 h at 70°C. Then, 5 μl of 2× DSN buffer (preheated to 70°C) and 0.25 Kunitz units of DSN enzyme were added and incubated at 70°C for 20 min. The DSN enzyme specifically degrades double-stranded molecules. The reaction was inactivated by adding 10 μl of DSN stop solution, and sterile water added to a final volume of 40 μl.

Following normalization, two rounds of PCR were performed using 1 μl of the normalization reaction as template. A shorter primer M1 (first 23 bases of the SMART IV oligonucleotide) was used in the first round of PCR with 15 amplification cycles using the same thermal cycling parameters as above; and an even shorter primer M2 (first 20 bases of the SMART IV oligonucleotide) was used in the second round of PCR for 15 amplification cycles of 95°C for 7 s, 64°C for 20 s, and 72°C for 5.5 min. Products were checked on a 1.1% agarose gel. The PCR products were quantified and 3 μg were used for treatment with proteinase K. All the subsequent procedures including proteinase K treatment, restriction digestion with *Sfi *I, size fractionation, and ligation followed the manufacture's instructions (Clontech). The cDNA was ligated to the pDNR-LIB vector. Electroporation (MicroPulser, Bio-Rad, Hercules, CA) was performed using DH12S electrocompetent cells following supplier's instructions (Invitrogen). A total of approximately 700,000 primary recombinant clones were obtained, and the library was amplified, titered, and stored in glycerol stocks in a -80°C freezer.

### Plasmid isolation and EST sequencing

Independent colonies were picked and grown for 20 h at 37°C in 1.2 ml LB broth containing 30 μg/ml chloramphenicol. Plasmid DNA was isolated using the Perfectprep Plasmid 96 Vacuum Direct Bind Kit from Eppendorf (Westbury, NY). Plasmids were stored at -20°C until usage. The cDNA inserts were directionally sequenced from the 5'-end of the cDNAs using universal M13(-21) primer and the BigDye terminator sequencing kit version 3.1 from Applied Biosystems (Foster City, CA) on a 3130XL DNA analyzer (Applied Biosystems).

### Sequence analysis

Base calling was performed using the Phred program [30,31] at quality cut-off set at Phred 20. Raw sequences were then imported into the Vector NTI Advance 10 software (Invitrogen) and were subjected to trimming of vector sequences and 5'adapter sequences using default settings. Afterwards, poly (A) tails were trimmed where necessary and sequences less than 100 bases were removed. Contigs were built in Vector NTI ContigExpress using default settings. All unique sequences were compared to the GenBank database using BLASTX in the non-redundant (NR), Swiss-Prot, and *Plasmodium falciparum *3D7 genome database. For comparison to the *Tetrahymena thermophila *SB210 genome, tBLASTx was used. The cut-off for sequence similarity used was E-value < 10^-5 ^for all analyses. Ciliate nuclear translation code was used in the BLAST searches. Search results from genome comparisons were summarized using a Venn diagram.

Gene ontology (GO) annotations were assigned using the program Blast2GO [48]. BLASTX results were loaded into the program and the default settings were used to assign GO terms to all unique sequences. From these annotations, pie charts were made using 2^nd ^level GO terms based on biological process, molecular function, and cellular component.

Putative secretory proteins and signal peptides were identified using both neural networks and hidden Markov model methods in SignalP 3.0 [43]. All 4,706 unique ESTs were used as the tester sequences. Open reading frames were predicted using both OrfPredictor [51] and BLASTX, with ciliate nuclear genetic code for ESTs of known genes and just OrfPredictor with unknown ESTs. The resulting deduced protein sequences from the ORFs were uploaded into SignalP 3.0. Sequences were identified as putatively secretory, predicted with signal peptides if both *D-score *in the neural network model and prediction probability in the hidden Markov model were significant.

Total lengths of all ESTs, G+C% content and simple repetitive elements were estimated using the Repeatmasker program [52].

### Accession numbers

All Ich EST sequences were submitted to the dbEST database of NCBI. Continuous accession numbers are from EG957858–EG966289.

## Authors' contributions

JWA, CA and ZJL planned the experiment and drafted the manuscript. HK, PL and PX provided technical assistance including cDNA library construction and sequencing support. PK and DX provided technical support and valuable Ich knowledge along with sample preparations. All were involved in final manuscript editing.

## Supplementary Material

Additional file 1 Supplemental Table 1, Excel spreadsheet; Table of *I. multifiliis *unique EST sequences; Provided information includes *I. multifiliis *BLASTX top hits to the non-redundant database in GenBank with unique EST name and accession numbers. Also included are significant protein domain comparisons to the Swiss-Prot database. Putative secretory proteins are highlighted. Click here for file

Additional file 2 Supplemental Table 2, Excel spreadsheet; Summary of BLAST searches of the Ich ESTs against *Tetrahymena thermophila *and *Plasmodium falciparum *genomes. Provided information includes *I. multifiliis *BLASTX top hits to the non-redundant database in GenBank with unique EST name, tBLASTx top hits to the *T. thermophila *genome, and BLASTX top hits to the *P. falciparum *genome sequences. This table correlates with the Venn diagram in figure 1. Click here for file

Additional file 3 Supplemental Table 3, Excel spreadsheet; Table of gene ontology (GO) profiles; Provided information includes unique EST name, accession numbers, BLASTX top hit, GO identification numbers and enzyme commission (EC) numbers. Click here for file
